# *NRG1* Genetic Variant Influences the Efficacy of Androgen-Deprivation Therapy in Men with Prostate Cancer

**DOI:** 10.3390/biomedicines9050528

**Published:** 2021-05-10

**Authors:** Shu-Pin Huang, Yei-Tsung Chen, Lih-Chyang Chen, Cheng-Hsueh Lee, Chao-Yuan Huang, Chia-Cheng Yu, Victor C. Lin, Te-Ling Lu, Bo-Ying Bao

**Affiliations:** 1 Department of Urology, Kaohsiung Medical University Hospital, Kaohsiung 807, Taiwan; shpihu@kmu.edu.tw (S.-P.H.); 1000660@ms.kmuh.org.tw (C.-H.L.); 2 Graduate Institute of Clinical Medicine, College of Medicine, Kaohsiung Medical University, Kaohsiung 807, Taiwan; 3 Department of Urology, Faculty of Medicine, College of Medicine, Kaohsiung Medical University, Kaohsiung 807, Taiwan; 4 Center for Cancer Research, Kaohsiung Medical University, Kaohsiung 807, Taiwan; 5 Department of Life Sciences and Institute of Genome Sciences, National Yang Ming Chiao Tung University, Taipei 112, Taiwan; yeitsungchen@ym.edu.tw; 6 Department of Medicine, Mackay Medical College, New Taipei City 252, Taiwan; lihchyang@mmc.edu.tw; 7 Department of Urology, College of Medicine, National Taiwan University Hospital, National Taiwan University, Taipei 100, Taiwan; cyhuang0909@ntu.edu.tw; 8 Department of Surgery, Division of Urology, Kaohsiung Veterans General Hospital, Kaohsiung 813, Taiwan; ccyu@vghks.gov.tw; 9 Department of Urology, School of Medicine, National Yang-Ming University, Taipei 112, Taiwan; 10 Department of Pharmacy, College of Pharmacy and Health Care, Tajen University, Pingtung 907, Taiwan; 11 Department of Urology, E-Da Hospital, Kaohsiung 824, Taiwan; ed102161@edah.org.tw; 12 School of Medicine for International Students, I-Shou University, Kaohsiung 840, Taiwan; 13 Department of Pharmacy, China Medical University, Taichung 404, Taiwan; lutl@mail.cmu.edu.tw; 14 Sex Hormone Research Center, China Medical University Hospital, Taichung 404, Taiwan; 15 Department of Nursing, Asia University, Taichung 413, Taiwan

**Keywords:** androgen-deprivation therapy, neuregulin, meta-analysis, prostate cancer, survival

## Abstract

Neuregulins (NRGs) activate receptor tyrosine kinases of the ErbB family, and play essential roles in the proliferation, survival, and differentiation of normal and malignant tissue cells. We hypothesized that genetic variants of NRG signalling pathway genes may influence treatment outcomes in prostate cancer. To test this hypothesis, we performed a comprehensive analysis to evaluate the associations of 459 single-nucleotide polymorphisms in 19 NRG pathway genes with cancer-specific survival (CSS), overall survival (OS), and progression-free survival (PFS) in 630 patients with prostate cancer receiving androgen-deprivation therapy (ADT). After multivariate Cox regression and multiple testing correction, we found that *NRG1* rs144160282 C > T is significantly associated with worsening CSS, OS, and PFS during ADT. Further analysis showed that low expression of *NRG1* is closely related to prostate cancer, as indicated by a high Gleason score, an advanced stage, and a shorter PFS rate. Meta-analysis of 16 gene expression datasets of 1,081 prostate cancer samples and 294 adjacent normal samples indicate lower *NRG1* expression in the former compared with the latter (*p* < 0.001). These results suggest that *NRG1* rs144160282 might be a prognostic predictor of the efficacy of ADT. Further studies are required to confirm the significance of *NRG1* as a biomarker and therapeutic target for prostate cancer.

## 1. Introduction

Prostate cancer is one of the major causes of mortality worldwide with an estimated 1.4 million new cases and 0.38 million deaths in 2020 [1]. Since the critical role of androgens in stimulating prostate cancer growth was established, androgen deprivation therapy (ADT) has become the most common concomitant therapy for prostate cancer [2]. Although most patients with prostate cancer initially respond to ADT, the duration of this response is highly variable. Once patients experience disease progression despite hormonal manipulation, the median survival period of patients with castration-resistant prostate cancer (CRPC) is 15–36 months depending on the tumour characteristics [3]. Currently, several clinicopathological factors, such as prostate-specific antigen (PSA), Gleason score, and cancer stage, have been concluded as predictors for the efficacy of ADT, but patient prognosis remains heterogeneous. Previously conducted epidemiological studies on twins suggest that prostate cancer is an inherited disease, with approximately 42% of its risk attributed to genetic factors [4]. To date, genome-wide association studies (GWAS) have identified more than 100 prostate cancer susceptibility loci [5]. However, these loci explain only 33% of the familial risk of prostate cancer, suggesting that a significant proportion of prostate cancer heritability remains undiscovered. Compared to GWAS, a hypothesis-driven pathway-based approach is advantageous as it avoids stringent multiple testing corrections and false negative results. 

Neuregulins (NRGs) are a subclass of the epidermal growth factor family, and are also known as heregulins, because they were initially identified while searching for Erb-B2 receptor tyrosine kinase 2 (ERBB2/HER2/NEU) activators [6]. Four *NRG* genes (*NRG1-4*) can be coded for more than 30 different protein isoforms using distinct promoters and alternative splicing [7]. NRGs principally act as ligands by binding to ERBB3/HER3 and ERBB4/HER4, and lead to the stimulation of diverse pathways, including mitogen-activated protein kinase (MAPK), protein kinase C, signal transducer and activator of transcription, and phosphatidylinositol-4,5-bisphosphate 3-kinase (PI3K)/Akt serine/threonine kinase (AKT) signalling pathways, resulting in the regulation of cell proliferation and development in multiple organs [8]. Recent genetic studies have shown that several germline variants of *NRG* genes are associated with the risks of developing thyroid and breast cancers [9,10]. An intronic variant of *NRG1* was also found to be associated with non-small cell lung cancer survival [11]. Moreover, a polymorphism in *NRG3* might lead to better overall survival in patients with stage IV epithelial ovarian cancer who are receiving first-line treatment [12]. However, the roles of genetic variants within the NRG signalling pathway in prostate cancer are unclear, and investigations into their functions may provide insight into the etiology and prognostic significance of the disease.

Since the NRG signalling pathway is considered to reflect the underlying biological processes of cancer development and progression, it is anticipated that single-nucleotide polymorphisms (SNPs) in NRG pathway genes may influence the response to cancer therapy. Therefore, we performed a pathway-based survival analysis to evaluate SNPs within the NRG pathway in a cohort of patients with prostate cancer treated with ADT.

## 2. Materials and Methods

### 2.1. Patient Response Evaluation

This study included 630 patients with prostate cancer who had been treated with ADT in three medical centres in Taiwan: National Taiwan University Hospital, Kaohsiung Medical University Hospital, and Kaohsiung Veterans General Hospital, as described previously [13,14]. The study was approved by the institutional review board of Kaohsiung Medical University Hospital (KMU-HIRB-2013132) in compliance with the Good Clinical Practice guidelines, and written informed consent was obtained from all participants. Clinicopathological information was collected from the patients’ medical records. Cancer-specific survival (CSS) was defined as the time from the initiation of ADT to the last follow-up or cancer-related death. Overall survival (OS) was defined as the time from the initiation of ADT to death attributable to any disease. Progression-free survival (PFS) was defined as the time from the initiation of ADT to disease progression or cancer-related death.

### 2.2. SNP Selection and Genotyping

Haplotype-tagging SNPs (htSNPs) covering 19 NRG pathway genes, including *NRG1-4*; *ERBB4*; PI3K catalytic subunits alpha, beta, and delta (*PIK3CA*, *PIK3CB*, and *PIK3CD*); the mechanistic target of rapamycin kinase (*MTOR*); *AKT1-3*; BCL2 associated agonist of cell death (*BAD*); glycogen synthase kinase 3 beta (*GSK3B*); cyclin dependent kinase inhibitors 1A and 1B (*CDKN1A* and *CDKN1B*); eukaryotic translation initiation factor 4E binding protein 1 (*EIF4EBP1*); and ribosomal protein S6 kinases B1 and B2 (*RPS6KB1* and *RPS6KB2*) and their five kb flanking regions were selected using the 1000 Genomes Project data for Han Chinese in Beijing, China and Southern Han Chinese with the Haploview 4.2 tagger algorithm [15]. Genomic DNA was extracted from peripheral lymphocytes and genotyping was performed using the Affymetrix Axiom Genotyping Arrays system (Thermo Fisher Scientific, Waltham, MA, USA) at the National Centre for Genome Medicine, Taiwan, as described previously [16]. SNPs with genotyping call rates < 0.9, minor allele frequency (MAF) < 0.02, and deviation from the Hardy–Weinberg equilibrium < 0.001 were excluded, leaving 459 htSNPs for further analyses.

### 2.3. Bioinformatics Analysis

HaploReg v4.1 was used to evaluate the functional significance of *NRG1* rs144160282 [17]. We used lymphoblastoid cell data from HapMap3 to evaluate the association of rs144160282 genotypes with the expression levels of *NRG1* [18]. The associations between *NRG1* expression and tissue types, Gleason score, stage, and survival outcome of prostate cancer were assessed using data from The Cancer Genome Atlas Prostate Adenocarcinoma (TCGA PRAD) [19] and the Oncomine [20] database.

### 2.4. Statistical Analysis

Statistical Product and Service Solutions version 19.0.0 (IBM, Armonk, NY, USA) was used for statistical analyses, and a two-sided *p* < 0.05 was considered significant. Multiple testing correction was applied to control the false-discovery rate (FDR, *q* values) under 0.20 [21]. We performed meta-analysis using RevMan 5.4.1 (Cochrane, London, UK), and a random-effect model was conducted due to potential heterogeneity between studies.

## 3. Results

The basic characteristics of the 630 patients involved in this study are presented in Table 1. After a median follow-up of 165.8 months, the median CSS, OS, and PFS values were found to be 135, 109, and 23 months, respectively. Age, clinical stage, Gleason score at diagnosis, PSA at ADT initiation, PSA nadir, and time to PSA nadir were all significantly associated with CSS, OS, and PFS (*p* ≤ 0.028).

**Figure 1:**
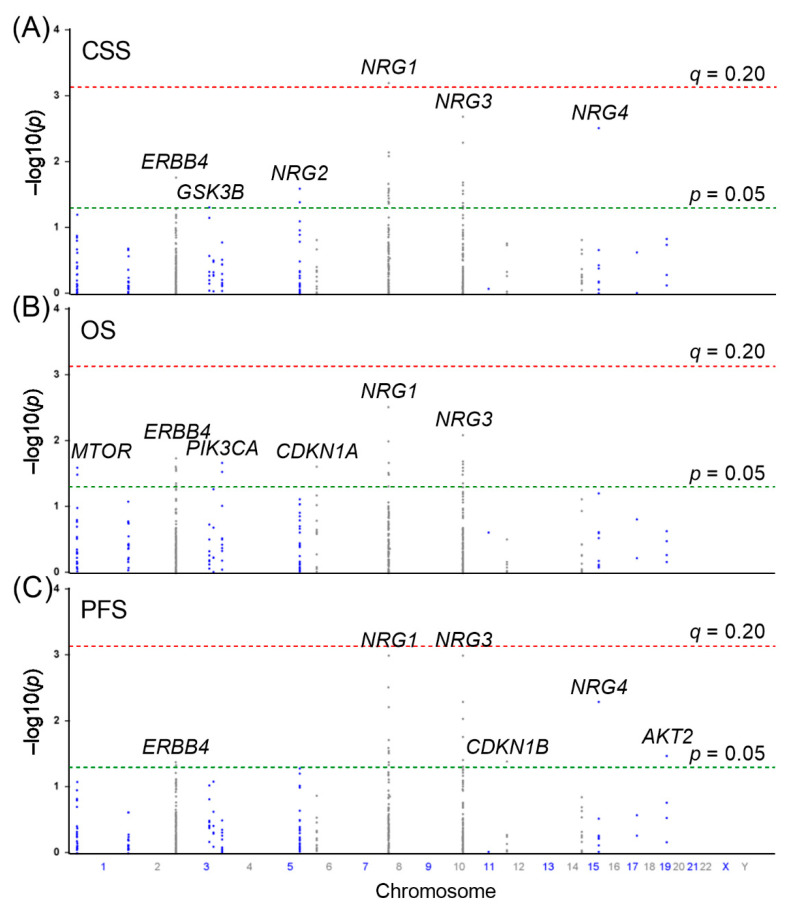
Manhattan plots of 459 single-nucleotide polymorphisms (SNPs) in 19 neuregulin (NRG) pathway genes with (**A**) cancer-specific survival (CSS), (**B**) overall survival (OS), and (**C**) progression-free survival (PFS) for patients with prostate cancer treated with androgen-deprivation therapy (ADT). The associations between SNPs and CSS, OS, and PFS are plotted as −log10 (*p*) values against their respective positions on the chromosomes. Genes are labelled if they contain associated SNPs, with *p* < 0.05. The red line denotes significance (*q* = 0.20) and the green line denotes *p* = 0.05.

**Figure 2:**
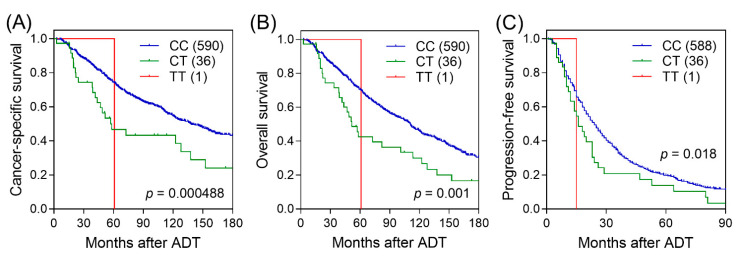
Kaplan–Meier curves estimating the associations of *NRG1* rs144160282 with (**A**) cancer-specific survival (CSS), (**B**) overall survival (OS), and (**C**) progression-free survival (PFS) in patients with prostate cancer under ADT. Values in brackets represent the number of patients.

We first performed Cox regression analysis to evaluate the associations between the 459 SNPs of the NRG pathway genes and the time of CSS, OS, and PFS during ADT. The results are summarized in a series of Manhattan plots (Figure 1). Twenty-eight, twenty-one, and twenty-six SNPs were individually associated with CSS, OS, and PFS, respectively, at *p* < 0.05 under the additive genetic model. After the multiple testing correction, only *NRG1* rs144160282 was significantly associated with CSS with FDR *q* < 0.20. Patients carrying the rare allele T of rs144160282 had worse CSS following ADT than those carrying the C allele (hazard ratio (HR) = 1.94, 95% confidence interval (CI) = 1.33–2.83, *p* = 0.00062, *q* = 0.168; Table 2, Figure 2A). Multivariate analysis with adjustment for clinical factors showed that *NRG1* rs144160282 remained significant as an independent prognostic factor for CSS (*p* = 0.018). Interestingly, patients carrying the T allele of *NRG1* rs144160282 also had worse OS and PFS in both univariate and multivariate analyses (Table 2, Figure 2B,C).

**Figure 3:**
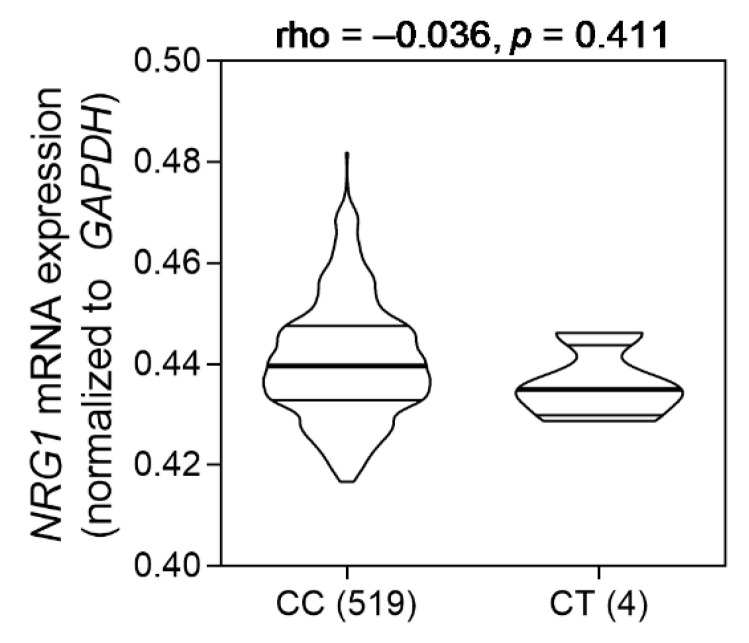
Correlation between rs144160282 genotypes and *NRG1* expression. Data were calculated using HapMap3 lymphoblastoid cell data. Values in brackets represent the number of samples.

Subsequently, we conducted functional annotation of rs144160282, which is an intronic SNP of *NRG1*, using HaploReg v4.1. The results showed that rs144160282 was located within a promoter and enhancer element due to the presence of histone modifications in this region in several tissues (Table 3). In addition, rs144160282 is likely to affect the DNase footprint and alter the Myf (myogenic differentiation 1, MYOD1) binding motif, indicating that rs144160282 was probably involved in the regulation of *NRG1* expression. Expression quantitative trait locus analysis was performed using HapMap3 lymphoblastoid cell data to assess the correlation of the rs144160282 genotype with the mRNA expression level of *NRG1*. The analysis suggested a trend of rs144160282 risk-allele T with reduced *NRG1* expression (correlation coefficient ρ = −0.036; Figure 3), but the correlation was not statistically significant, possibly due to the limited number of individuals (*n* = 4) carrying the T allele.

**Figure 4:**
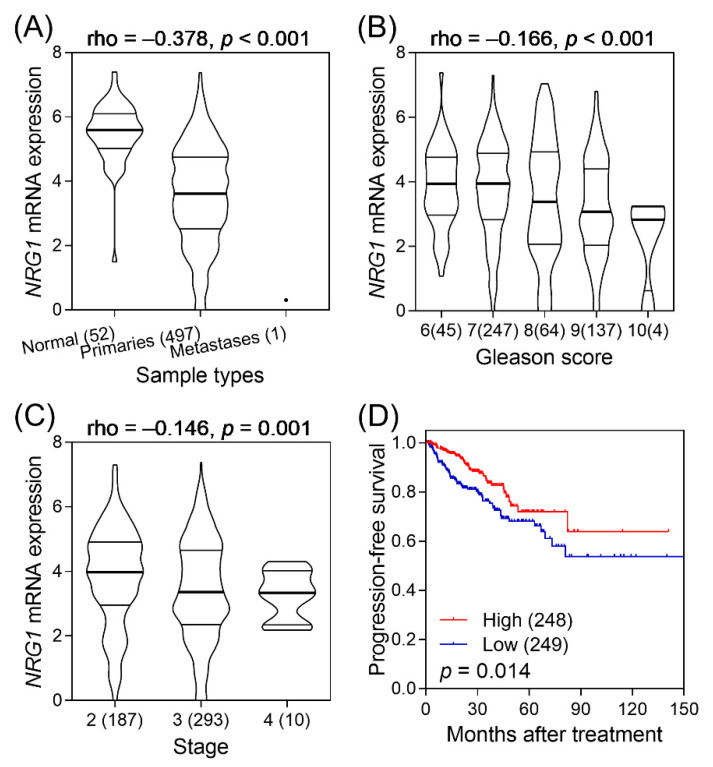
Clinical significance of *NRG1* in prostate cancer. (**A**) Down-regulation of *NRG1* expression in The Cancer Genome Atlas Prostate Adenocarcinoma samples. A negative correlation between the expression of *NRG1* and (**B**) the Gleason score and (**C**) the tumour stage was found. (**D**) Patients with low *NRG1* expression exhibited reduced PFS compared to patients with high *NRG1* expression. Rho (ρ)—Spearman’s rank correlation coefficient. Values in brackets represent the number of patients.

**Figure 5:**
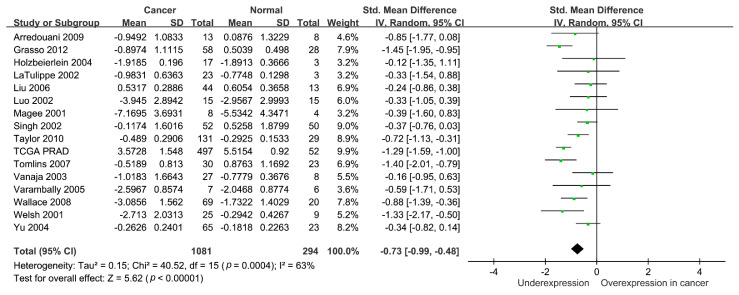
Meta-analysis of *NRG1* expression levels between tumor and normal tissues in 16 independent prostate cancer studies. *NRG1* showed lower expression in prostate cancer than in normal tissues. SD, standard deviation. IV, inverse variance. CI, confidence interval. Std, standardized.

The clinical relevance of *NRG1* in prostate cancer was further assessed using a TCGA PRAD dataset. As shown in Figure 4, the expression level of *NRG1* mRNA decreased in prostate cancer as compared to adjacent noncancerous tissues (*p* < 0.001). Further, it also decreased with increasing Gleason score (*p* < 0.001) and with higher tumor stage (*p* < 0.001). Lower *NRG1* expression was associated with significantly poor PFS in patients with prostate cancer (*p* = 0.014). Furthermore, a meta-analysis of 16 publicly available prostate cancer gene expression datasets, comprising a total of 1,081 prostate cancer cases and 294 adjacent normal tissues revealed downregulated *NRG1* expression in prostate cancer compared with that of adjacent normal tissues (standardized mean difference = −0.73, 95% CI = −0.99 to −0.48, *p* < 0.001; Figure 5). Together, these findings indicate that *NRG1* has potential anti-tumorigenic effects in prostate cancer.

## 4. Discussion

This is the first study to systematically assess the genetic variants of the NRG pathway in relation to the efficacy of ADT in patients with prostate cancer. We found that *NRG1* rs144160282 was associated with CSS, OS, and PFS during ADT, even after adjusting for multiple testing and clinical predictors. Moreover, downregulation of *NRG1* is associated with prostate cancer progression and decreased PFS in patients.

The risk variant rs144160282 lies within the *NRG1* gene, a ligand of ERBB3/HER3 and ERBB4/HER4 possibly involved in ERBB signalling and related to tumorigenesis. *In silico* analysis revealed that rs144160282 is located in the intron region with some promoter and enhancer histone marks, DNase hypersensitive sites, and altered MYOD1 transcription factor binding motif. MYOD1 was found to be upregulated in CRPC, and knockdown of MYOD1 impaired castration-resistant LNCaP/R cell proliferation and induced apoptosis on androgen depletion [22]. We found that the mRNA expression of *NRG1* was lower in prostate cancer than in normal tissues, and the low expression levels were associated with shorter patient PFS. Although the rs144160282 risk-allele T showed a trend of reduced *NRG1* expression, the correlation was not significant due to the limited number of individuals with the T allele. Therefore, the mechanism by which *NRG1* rs144160282 affects the efficacy of ADT in patients with prostate cancer needs to be further investigated.

Human NRGs have more than 30 different isoforms that can be grouped into six types based on the distinct *N*-terminus, and the expression of NRG isoforms differs significantly in a tissue-specific manner [7]. Interestingly, most of the NRG isoforms are synthesized as transmembrane molecules acting on cells via physical contact (juxtacrine), but they can also be solubilized by cell surface proteases and act via paracrine and autocrine modes [23]. The epidermal growth factor-like domain of NRGs binds to and induces dimerization of ERBB receptors. Activation of ERBB receptors is often associated with aggressive forms of tumor and poor patient prognosis, whereas ERBB4 signaling has been found to have cell growth inhibiting properties [24]. In addition, a low concentration of NRGs has been found to be mitogenic in human breast cancer cells, whereas a high concentration leads to cell differentiation and growth inhibition [25]. Due to the complexity, solubility or membrane-bound nature of isoforms, tissue distribution, and receptor availability of NRGs, the tissue-specific effects of NRG-induced cell proliferation and differentiation may depend on different cellular contexts. 

The role of *NRG1* appears to be paradoxical in cancer. NRG1 could be oncogenic, as it binds to ERBB receptors and activates downstream cell proliferation- and survival-related pathways, such as MAPK and PI3K/AKT. In addition, fusions of the *NRG1* gene with various partner genes, such as the CD74 molecule, solute carrier family 3 member 2, and unc-5 netrin receptor D, have been identified in a wide range of cancer types, although their frequency is low with only 82 such examples out of 44,570 tumors (0.2%) [26]. Almost all *NRG1* gene fusions contain the receptor-binding domain that induces receptor dimerization and subsequent ERBB pathways, resulting in abnormal cell proliferation [27]. Therefore, ERBB-targeted treatments, such as the monoclonal antibody zenocutuzumab and the small molecule afatinib, have been evaluated for their anticancer efficacy in patients with *NRG1* fusion-positive cancers. Partial responses to afatinib treatment were achieved for up to 12 months in patients with lung adenocarcinoma [28,29], and up to 5.5 months in patients with pancreatic ductal adenocarcinoma [30]. However, while normal epithelial cells produce significant amounts of *NRG1* and its receptors, many cancer cell lines have reduced expression of *NRG1* [31,32]. Genetic analyses have demonstrated that the short arm of chromosome 8 (8p) is frequently lost in epithelial cancers, including prostate, breast, and colon cancers [33]. Mapping the loss of 8p in cancer cells revealed that almost all the translocation breaks were proximal to *NRG1*, making it a candidate tumor suppressor gene [34]. The absence of *NRG1* expression in tumours has also often been associated with DNA methylation at the CpG island around the transcription start site of *NRG1* [31]. Reducing *NRG1* expression by small interfering RNA increased cell proliferation in both normal and human breast cancer cells [31], whereas expression of *NRG1* induced apoptosis via the downregulation of the BCL2 apoptosis regulator [35]. Immunohistochemical studies of clinical prostatectomy specimens demonstrated that the NRG and ERBB4 receptor proteins are strongly expressed in normal prostate luminal cells but not in prostate cancer [36]. Treatment with NRGs significantly reduces the growth of androgen-sensitive prostate cancer cells [36], but increases CWR-R1 CRPC cell proliferation [37]. Similarly, increased membranous NRG expression was found to be correlated with improved prognosis in hormone-naive prostate cancer, but had no effect in CRPC [38]. In line with our meta-analysis, NRG1 protein was shown to express in 100% (3 of 3) of normal prostate tissues with moderate cytoplasmic/membranous expression, whereas the expression was undetectable in all (0 of 12) prostate cancer tissues from The Human Protein Atlas, confirming that NRG1 protein expression is downregulated in prostate cancer. Although this evidence supports our findings that *NRG1* may have a protective effect in prostate cancer and its loss may worsen patient prognosis, the exact mechanisms need to be further investigated.

In our multivariate analysis, *NRG1* rs144160282 retained its association with clinical outcomes after ADT while known clinicopathological risk factors (age, stage, Gleason score at diagnosis, PSA at ADT initiation, PSA nadir, and time to PSA nadir) were controlled. This new genetic information adds predictive value above and beyond conventional factors. Among the strengths of this study, well-validated outcome parameters and complete follow-up details of the participants are available from high-quality national registries for cancer-related and all-cause mortality evaluations. HtSNPs were selected to ensure thorough coverage across each haplotype block of all NRG pathway genes. Although we observed some associations between *NRG1* and prostate cancer supported by multiple functional data, the exact molecular mechanisms remain to be elucidated. In particular, as the expression quantitative trait loci analysis did not show a correlation between the rs144160282 genotypes and the expression levels of *NRG1*, further fine-mapping studies are required to identify the causal variants that can corroborate the present findings. The generalizability of the present study is limited, because all the participants in this study are Taiwanese. Furthermore, rs144160282 seems to be East Asian-specific, since it is found to be monomorphic in all other populations, according to 1000 Genomes Project data.

## 5. Conclusions

Genetic association studies have been widely used to facilitate gene hunting or locate genes associated with complex diseases such as cancer. In this study, we found that *NRG1* rs144160282 could independently predict CSS, OS, and PFS following ADT among patients with prostate cancer. Since downregulation of *NRG1* in prostate cancer was associated with a higher Gleason score, more advanced-stage tumours, and worsening patient PFS, it can be stated that *NRG1* plays a tumor suppressive role in prostate cancer. Larger cohort studies and additional functional experiments are warranted to validate our findings.

## Figures and Tables

**Table 1:** Clinicopathologic characteristics of the study population.

Characteristics	*n* (%)	CSS ^a^			OS ^a^			PFS ^a^		
		Events, *n*	Median, Months	*p*	Events, *n*	Median, Months	*p*	Events, *n*	Median, Months	*p*
Total, *n* (%)	630	314	135		413	109		518	23	
**Age at Diagnosis, Years**										
Median (IQR)	73 (67–79)									
<74	344 (54.7)	168	154	0.014	201	128	<0.001	295	20	<0.001
≥74	285 (45.3)	145	120		211	86		222	29	
**PSA at ADT Initiation, ng/mL**										
Median (IQR)	34.5 (11.25–129)									
<35	307 (50.6)	115	196	<0.001	167	138	<0.001	245	26	0.028
≥35	300 (49.4)	187	88		232	72		256	19	
**PSA Nadir, ng/mL**										
Median (IQR)	0.14 (0.01–1.16)									
<0.15	314 (50.7)	109	202	<0.001	167	159	<0.001	243	34	<0.001
≥0.15	305 (49.3)	200	65		239	59		270	15	
**Time to PSA Nadir, Months**										
Median (IQR)	11 (5–20)									
<12	323 (52.2)	177	96	<0.001	216	77	<0.001	276	12	<0.001
≥12	296 (47.8)	132	162		190	123		237	36	
**Clinical Stage at Diagnosis**										
T1/T2	187 (29.9)	70	NR	<0.001	103	138	<0.001	144	26	<0.001
T3/T4/N1	205 (32.8)	81	196		119	138		162	30	
M1	233 (37.3)	162	63		189	59		209	16	
**Gleason Score at Diagnosis**										
2–6	188 (30.6)	81	185	<0.001	112	133	<0.001	144	28	0.001
7	194 (31.6)	84	184		115	121		164	25	
8–10	232 (37.8)	143	73		177	63		196	18	

Abbreviations: CSS, cancer-specific survival; OS, overall survival; PFS, progression-free survival; IQR, interquartile range; PSA, prostate-specific antigen; NR, not reached. ^a^ With a median follow-up of 165.8 months. Subtotals do not sum to 630 due to missing data.

**Table 2:** Association of *NRG1* rs144160282 with CSS, OS, and PFS in prostate cancer patients receiving ADT.

Genotype	Frequency	CSS	HR (95% CI)	*p*	*q*	HR (95% CI) ^a^	*p* ^a^		
CC/CT/TT	591/36/1	286/25/1	1.94 (1.33–2.83)	0.00062	0.168	1.60 (1.08–2.35)	0.018		
**OS**	**HR (95% CI)**	***p***	**HR (95% CI) ^a^**	***p*^a^**	**PFS**	**HR (95% CI)**	***p***	**HR (95% CI) ^a^**	***p*^a^**
381/30/1	1.78 (1.26–2.52)	0.001	1.57 (1.10–2.24)	0.014	482/43/1	1.49 (1.06–2.08)	0.021	1.16 (0.83–1.63)	0.375

Abbreviations: CSS, cancer-specific survival; OS, overall survival; PFS, progression-free survival; ADT, androgen deprivation therapy; HR, hazard ratio; CI, confidence interval. ^a^ Adjustment for age, stage, Gleason score at diagnosis, PSA at ADT initiation, PSA nadir, and time to PSA nadir.

**Table 3:** Functional annotation of *NRG1* rs144160282.

Reference Allele	Alternate Allele	AFR Frequency	AMR Frequency	ASN Frequency	EUR Frequency	Variant Type	Promoter Histone Marks	Enhancer Histone Marks	DNAse	Motifs Changed
C	T	0.00	0.00	0.02	0.00	intronic	BLD	FAT, BRST, MUS, LNG, VAS, BONE	ESDR, SKIN, SKIN, LNG, MUS, MUS, MUS, SKIN, SKIN	Myf

Abbreviations: AFR, Africa; AMR, America; ASN, Asia; EUR, Europe.

## Data Availability

The data presented in this study are available on request from the corresponding author.
